# Satellite Fault Diagnosis Using Support Vector Machines Based on a Hybrid Voting Mechanism

**DOI:** 10.1155/2014/582042

**Published:** 2014-08-12

**Authors:** Hong Yin, Shuqiang Yang, Xiaoqian Zhu, Songchang Jin, Xiang Wang

**Affiliations:** ^1^College of Computer, National University of Defense Technology, Changsha 410073, China; ^2^Xiangyang School for NCOs, Xiangyang 441118, China

## Abstract

The satellite fault diagnosis has an important role in enhancing the safety, reliability, and availability of the satellite system. However, the problem of enormous parameters and multiple faults makes a challenge to the satellite fault diagnosis. The interactions between parameters and misclassifications from multiple faults will increase the false alarm rate and the false negative rate. On the other hand, for each satellite fault, there is not enough fault data for training. To most of the classification algorithms, it will degrade the performance of model. In this paper, we proposed an improving SVM based on a hybrid voting mechanism (HVM-SVM) to deal with the problem of enormous parameters, multiple faults, and small samples. Many experimental results show that the accuracy of fault diagnosis using HVM-SVM is improved.

## 1. Introduction

With the rapid development of the aerospace engineering, the system structure of satellite has become more and more complex, but the requirements of reliability and safety are also getting higher. However, due to the complexity of the space environment and the testing limitations of satellite, abnormal operation to the satellite or system failure problem often appears. The satellite fault diagnosis has an important role in improving the reliability, safety, and availability of the satellites and it has become the focus in the aerospace research.

Many methods for satellite fault diagnosis have been extensively studied, and these methods can be mainly divided into two categories. One approach is to use the model-based diagnosis for aerospace systems [1]. Another approach is the data-driven approach, also known as the data mining approach or the machine learning approach, which uses historical data to automatically learn a model of system behavior [2]. In model-based approaches, the Kalman filter, also known as linear quadratic estimation (LQE), is quite popular [3]. Although the model-based techniques have good performance in real-time fault diagnosis, their reliability will be decreased when the system nonlinearities, complexity, and modeling uncertainties increase.

Data-driven approaches mostly rely on real-time or historical data collected from the sensors and measurements, so they do not need a detailed mathematical model of the satellite. Many people made lots of contributions in this area. Park et al. applied the BEAM (Beacon-based exception analysis for multimissions) system to fault diagnosis in space shuttle main engine data [4]. They used the dynamical invariant anomaly detector (DIAD) to look for anomalies with a series of measurements observed over time. Schwabacher used two unsupervised anomaly detection algorithms, Orca and GritBot, to diagnose faults with data from two rocket propulsion systems [5]. Iverson's inductive monitoring system (IMS) [6] is another unsupervised learning system for fault diagnosis. It used an algorithm to cluster the nominal data into classes which represented different modes of the system. Ogaji [7] has extended multiple neural networks to isolate component and sensor faults using a cascaded network. Widodo and Yang [8] discussed the effectiveness of support vector machine (SVM) in the machine condition monitoring, and the experiment shows that the SVM is of high accuracy in the faults classification [9].

Most machine learning methods, including pattern recognition and neural networks, need sufficient and high quality sample data. To fault diagnosis, that is to say, the data need to cover all the failure modes, and the similar modes can not be of contradiction. But with the level of manufacture growing, the failure rate of satellite is reduced, so these algorithms are not good at the satellite fault diagnosis.

As mentioned above, satellite fault diagnosis is limited by the conditions of space environment, and large number of fault samples for training is not always obtainable in practice. Therefore, processing the small samples and being of good generalization are great significance for satellite fault diagnosis. SVM [10] developed by Vapnik is based on the minimum structural risk theory and has been widely applied since the 90s in various fault diagnosis and classification problems. It is very useful for the small samples and is characterized by good generalization ability. SVM provides viable tools to deal with nonlinear problems, and, to complex and nonlinear dynamical systems, it is of great flexibility and capability. Many improving algorithms for SVM also have been proposed. Compared with standard SVM method using inequality constraints, Suykens proposed LS-SVM [11]. Through the method, the second norm of the error becomes the optimization goal of the loss function; thus, the solution of the quadratic programming problem has been transformed into linear equations. Monroy et al. proposed a semisupervised approach [12] consisting of different methods such as Gaussian mixture models (GMM), independent component analysis (ICA), Bayesian information criterion (BIC), and SVM, and it is effectively applied to the entire set of the Tennessee Eastman process (TEP) faults. Combining supervised or unsupervised learning methods with SVM is a research hotspot of improving the performance of fault diagnosis, and many algorithms for optimizing traditional SVM have also shown improved performance. From this idea, we proposed a novel method for satellite fault diagnosis, called SVM based on hybrid voting mechanism (HVM-SVM). Considering the characteristics of small sample data, multiple faults, and enormous parameters, the main contribution of this paper is to improve the performance of SVM using a hybrid voting mechanism. The experimental results show that the classification accuracy of HVM-SVM to multiple faults has been enhanced.

The rest of this paper is organized as follows.  Section 2 outlines the problem of satellite fault diagnosis.  Section 3 contains the solution method.  Section 4 introduces the hybrid voting mechanism. The experimental results are discussed in Section 5. We conclude the paper in Section 6.

## 2. The Problem of Satellite Fault Diagnosis

For satellite fault diagnosis, there will be two problems that needed to be considered: multiple parameters and multiple faults. The satellite is a so complex system that it needs many parameters of components to record the running states. Take the remote sensing data of satellite for example; the number of parameters is more than two thousand. It is obvious that every fault can not be connected with all the thousands of parameters. So how to confirm the mapping relation between the faults and parameters is the first problem. Usually the fault can be divided into two categories:* single fault* and* multiple faults*. In the single-fault mode, it is assumed that there exists only one fault at any time in the system. Frequent testing and maintenance are needed to make sure of the above condition and it will lead to interpretation of uncertainties. However, single-fault assumption is not unreasonable in many real applications such as tolerant system and space-based system, where frequent testing and maintenance are not possible. Since the single fault assumption can lead to incorrect or failed diagnoses when multiple faults occur, the multiple faults diagnosis is more important. However, for the number of candidates faults growing exponentially, multiple faults diagnosis will be a challenging problem. In addition, multiple faults in dynamic systems like satellite may be hard to detect, because interactions among fault effects can obscure the fault signatures. The problem of multiple faults diagnosis can be described as follows. We denote the faults set by *F* = {*f*_1_, *f*_2_,…, *f*_*k*_} and the set of all measurements of parameters set *P* = {*p*_1_, *p*_2_,…, *p*_*n*_} by *M* = {*m*_1_, *m*_2_,…, *m*_*n*_}. For *f* ∈ *F* and *m* ∈ *M*, *σ*(*f*, *m*) represents the fault signature of *m* when the fault *f* occurred.

**Definition 1 (fault associativity).**

For all *f*_*i*_ ∈ *F*, when a *M*′ = (*m*_*i*_, *m*_*j*_,…*m*_*k*_)⊆*M* of the parameters set *P*′ = {*p*_*i*_, *p*_*j*_,…, *p*_*k*_}⊆*P* has exceeded a threshold set *ε*′ = {*ε*_*i*_, *ε*_*j*_,…, *ε*_*k*_}, the fault *f*_*i*_ will occur, and it means that the *f*_*i*_ is associated with the parameters set *P*′.

**Definition 2 (fault distinguishability).**

Given a measurements set *M*, if ∃*m* ∈ *M*, *σ*(*f*_*i*_, *m*) ≠ *σ*(*f*_*j*_, *m*), then the two faults *f*_*i*_, *f*_*j*_ ∈ *F* are distinguishable.

**Definition 3 (fault diagnosability).**

If, for all *f*_*i*_, *f*_*j*_ ∈ *F*, *f*_*i*_ and *f*_*j*_ are distinguishable, then the system is fault diagnosable.

**Definition 4 (multiple faults diagnosability).**

Given multiple faults *F*′ = {*f*_*i*_, *f*_*j*_,…, *f*_*k*_}⊆*F*, the elements of faults set with measurements signatures are denoted by *σ*(*F*′) = {*σ*(*F*′, *m*_1_), *σ*(*F*′, *m*_2_),…, *σ*(*F*′, *m*_*n*_)}, which satisfied the following: (for all *F*_*a*_′, *F*_*b*_′ ∈ *F*′) (∃*m* ∈ *M*)*σ*(*F*_*a*_′, *m*) ≠ *σ*(*F*_*b*_′, *m*).

**Figure 1:**
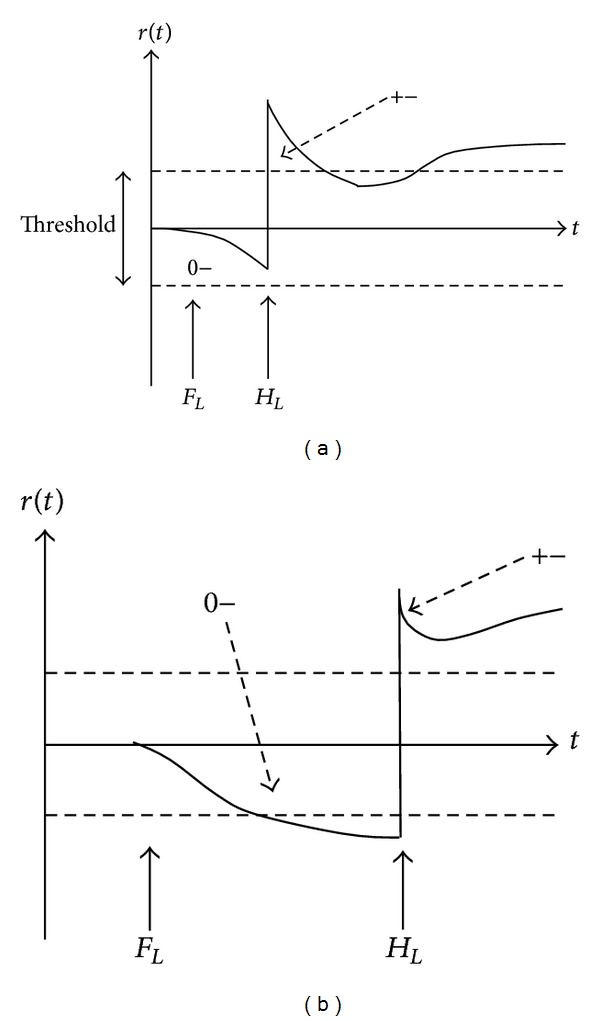
(a) *H*_*L*_ occurs close enough to *F*_*L*_; (b) *H*_*L*_ occurs after *F*_*L*_ (0− and 0+ represent that the value of parameter decreases and increases; ± represents that the value is first increased and then decreased).

That is, if the system is diagnosable, there is a unique fault which consisted with the deviations of some measurements. There are two reasons in the fact that multiple faults diagnosis is more complex than single-fault diagnosis. First, the effects of a fault would be masked or compensated by another fault. For example, the fault *F*_*L*_ may occur, causing deviations of 0− on *α*, 0+ on *β*, and 0− on *λ*. However, if *F*_*R*_ occurred concurrently, causing reverse deviations of 0+ on *α*, 0− on *β*, and 0+ on *λ*, then the two faults may not be distinguished. Second, the same multiple faults can be manifested in different ways. For example, fault set {*F*_*L*_, *H*_*L*_} will cause 0− or ± on *α*, depending on which fault occurs first and on the fault delays in system. From Figure 1, the *F*_*L*_ occurs first, the effect of 0− or ± will happen depending on how soon *H*_*L*_ occurs after *F*_*L*_. If *H*_*L*_ occurs close enough to *F*_*L*_, the 0− effect caused by *F*_*L*_ may not be detected.

## 3. Solution Methodology

In Section 2, it is pointed out that some faults are very difficult to find due to the interactions between these parameters. So it needs a comprehensive judgment method which may provide more complementary information about the faults. In this section, we will integrate the diagnosis results from faults associativity, SVM, and combining classifier to improve the accuracy of satellite fault diagnosis.

### 3.1. Fault Associativity

As described in Definition 1, the fault associativity is about the relation between fault and its corresponding parameters set. For any fault, using all the parameters of modeling, the accuracy of the fault diagnosis will be decreased. So how to confirm the mapping relations between faults and parameters is a key step for satellite faults diagnosis. Sometimes, the confirmation can be accomplished by domain experts. However, it is unreasonable to consider so many parameters, especially for the unknown faults. Based on rough sets [13], a method is used to improve the choosing of parameters.

An information system *S* can be represented as an ordered quaternion, *S* = 〈*U*, *R*, *V*, *f*〉, which consists of the following: *U* = {*x*_1_, *x*_2_,…} is a nonempty, finite set called the universe; *R* = *C* ∪ *D* is a nonempty, finite set of all attributes, in which *C* is the condition attributes set and *D* is the decision attributes set, *C*∩*D* = *ϕ*; *V* = ⋃_*r*∈*R*_ *V*_*r*_ is a set of some attributes, where *V*_*r*_ is called the domain of *r*; for *a* ∈ *R*, *x*_*i*_ ∈ *U*, *f*(*x*_*i*_, *a*) is the value of *x*_*i*_ in attribute *a*. In the following fault diagnosis table, the columns are labeled by attributes and rows by objects (the classes of faults), and *U* = {1,2, 3,4, 5,6} and *R* = {*a*, *b*, *c*, *d*, *e*}.

For every subset of attributes *B*⊆*R*, define an indiscernibility binary relation IND(*B*):

(1) $$\begin{array}{c} \text{IND}\left(B\right)=\left\{\left(x,y\right)\;|\;\left(x,y\right)\in U^{2},\;\forall b\in B\left(b\left(x\right)=b\left(y\right)\right)\right\}, \end{array}$$

where IND(*B*) is an equivalence relation and

(2) $$\begin{array}{c} \text{IND}\left(B\right)=\underset{b\in B}{⋂}\text{IND}\left(\left\{b\right\}\right). \end{array}$$

Objects *x*, *y* satisfying the relation IND(*B*) are indiscernible by attributes from *B*. Consider the subset *B* = {*a*, *b*, *c*} in Table 1, and then IND({*a*}) = ({1,3, 4,5, 6}, {2}); IND({*B*}) = ({1,3}, {2}, {4}, {5,6}).

**Definition 5 (discernibility matrices).**

Given *S* = 〈*U*, *R*, *V*, *f*〉, *U* = {*x*_1_, *x*_2_,…, *x*_*n*_}, *R* = *C* ∪ *D*, the subset *C* = *a*_*i*_∣*i* = 1,…, *m*, and *D* = {*d*}, *a*_*i*_(*x*_*i*_) is the value of *x*_*i*_ in attribute *a*_*i*_. The element of a discernibility matrix of *B* is defined as

(3) $$\begin{array}{c} \left(C_{ij}\right)=\{\begin{array}{cc} \left\{a_{k}\;|\;a_{k}\in B\cap a_{k}\left(x_{i}\right)\neq a_{k}\left(x_{j}\right)\right\}, & d\left(x_{i}\right)=d\left(x_{j}\right)\\ 0,d\left(x_{i}\right)\neq d\left(x_{j}\right) & i,j=1,\ldots ,n. \end{array} \end{array}$$

As mentioned above, in order to find the fault associativity, the parameter reduction is necessary. Supposing a parameters subset *P*⊆*R*, if *Q*⊆*P* is independent and IND(*P*) = IND(*Q*), then *Q* is called the parameter reduction of *P*, that is red(*P*) = {*Q*}. The parameter reduction algorithm based on discernibility matrix is as in Algorithm 1.

It can be seen from the reduction algorithm that the results can have multiple reductions and here the red(*P*) is the candidate set of fault associativities. Which red(*P*) will be chosen for a fault depended on the highest accuracy of the fault diagnosis model trained by *P*_*i*_. Supposing the faults set *F* = {*f*_1_, *f*_2_, *f*_3_}, the red(*P*) = {*P*_1_, *P*_2_, *P*_3_}, model *M*_1_ is trained using the parameter set *P*_1_, model *M*_2_ is trained using the parameter set *P*_2_ and model *M*_3_ is trained using the parameter set *P*_3_. The accuracy of model for the fault is defined as *A*(*M*_*i*_, *P*_*i*_, *f*_*j*_). So the corresponding parameters set of fault *f*_*j*_ can be defined as

(4) $$\begin{array}{c} f_{j}\;\text{red}\left(P\right)=\left\{P_{i}\;\;\text{in}\;\;\underset{i=1,2,\ldots ,m}{\max}\left(A\left(M_{i},P_{i},f_{j}\right)\right)\right\}. \end{array}$$

For example, if *A*(*M*_1_, *P*_1_, *f*_1_) is higher than *A*(*M*_2_, *P*_2_, *f*_1_) and *A*(*M*_3_, *P*_3_, *f*_1_), the red(*P*) = *P*_1_ will be selected as the parameters set related to fault *f*_1_.

### 3.2. The Fault Diagnosis Based on SVM

SVM is a technique used to train the classifiers based on the structural risk minimizations concept, and it has been widely applied in fault diagnosis. A fundamental knowledge about the classical SVM will be presented firstly.

**Figure 2:**
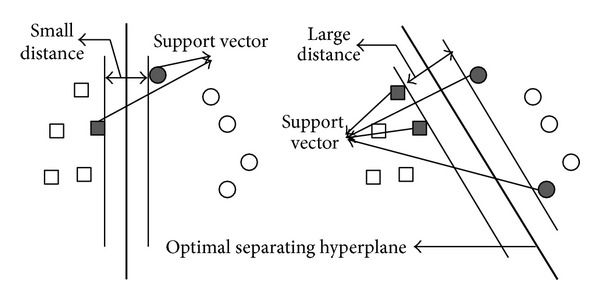
An example of optimal separating hyperplane.

SVM is a binary classifier which can be used to classify data into two classes: positive and negative. Supposing a set of points with two classes, SVM establishes a hyperplane that separates the majority of positive points from the negative points and maximizes the distance between the two classes to this hyperplane. The maximum distance hyperplane is also called the optimal separating hyperplane. The nearest points of two classes to hyperplane are employed to define the support vectors.  Figure 2 shows an example of optimal separating hyperplane of two classes.

Supposing a known training set {*x*_*i*_, *y*_*i*_} (*i* = 1,…, *n*), *x* ∈ *R* ^*n*^, *y* ∈ {−1, 1}, *x*_*i*_ is the input vector, and *y*_*i*_ is the required classification. The SVM is to estimate a function *f* that can separate the given data {*x*_*i*_, *y*_*i*_}. The optimal hyperplane is defined as

(5) $$\begin{array}{c} w^{T}x+b=0, \end{array}$$

where *w* ∈ *R* ^*n*^ is a vector of weights and *b* is a scalar bias term. The *w* and *b* are used to describe the position of the hyperplane. A vector *x*_*i*_ with the same class of *y*_*i*_ must satisfy the equation

(6) $$\begin{array}{c} y_{i}\left(wx_{i}+b\right)\geq 0,\;y_{i}\in \left\{-1,1\right\}. \end{array}$$

To satellite fault diagnosis, most patterns are not linearly separated. In order to decrease the computational efforts of the support machines, the SVM constructs an optimal separating hyperplane in this higher dimensional space called feature space by choosing a nonlinear mapping a priori. A positive slack variable *ε*_*i*_ for every training sample is defined to obtain a hyperplane with larger distance. This also permits some samples to be misclassified. So searching the optimal hyperplane can be obtained as a solution to the following constrained quadratic optimization problem:

(7) Minimise (12||w2||−C∑i=1Nεi)Subject  to yi(wxi+b)≥1−εi,i=1,…,n,

where *C* is the regularization parameter that determines the balance between the maximization hyperplane and minimization classification error. If 0 ≤ *ε*_*i*_ ≤ 1, it means that *x*_*i*_ is on the right side of the hyperplane, and the pattern is classified correctly. If *ε*_*i*_ > 1, it means that *x*_*i*_ is on the wrong side of the hyperplane.

The basic form of SVM is a binary classifier which separates a set of positive examples from a set of negative examples, also called dichotomies. For more than two classes, unfortunately, there is no unique method for SVM to deal with multiple faults. The general approaches adapting SVM to multiple classes are to reduce the problem of multiclass to a set of binary problems. One method is to construct *m* binary classifier where the *m* is the number of classes. It is called the one-against-all for every binary classifier separates one class from all the other classes. Using the method to classify a new sample, each binary classifier generates a class and the result with the highest confidence is chosen finally. Another strategy constructs *m*(*m* − 1)/2 binary classifiers; each of them separates only two classes. For example, to the faults set *F* = {*f*_1_, *f*_2_, *f*_3_}, there are 3 × (3 − 1)/2 = 3 classifiers constructed to classify binary seta {*f*_1_, *f*_2_}, {*f*_1_, *f*_3_}, and {*f*_2_, *f*_3_}, respectively. The majority voting strategy is adapted to classify a new sample in which the *m*(*m* − 1)/2 SVM classifiers will vote for each class, and the class with the maximum votes is selected. This method is certainly more efficient than one-against-all, but is has a major drawback. That is, each classifier model is trained by the data only from two classes and not considering the fault associativity, but, in the fault diagnosis phase, the outputs using data may be from any class [14]. To solve the problem, the combining classifiers strategy is used to obtain the synthesis decision.

### 3.3. Combining Classifiers

**Figure 3:**
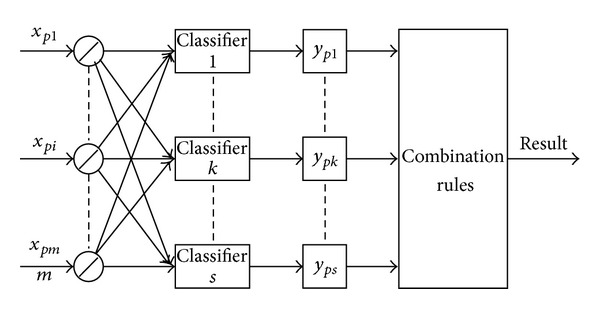
An example of combining classifier.

As different classifiers may offer complementary information about the fault to be classified, combining classifiers, in an efficient way, can achieve better classification results than any single classifier. To multiple faults diagnosis, the ultimate goal of combining classifiers is to achieve the best possible classification performance of the faults. The combining classifier is to combine the outputs of multiple classifiers into one classification result according to some rules. An example of combining classifier is shown in Figure 3. There are many combination rules for combining classifier, such as* max rule*,* min rule*,* median rule*, and* majority vote rule*.

**Figure 4:**
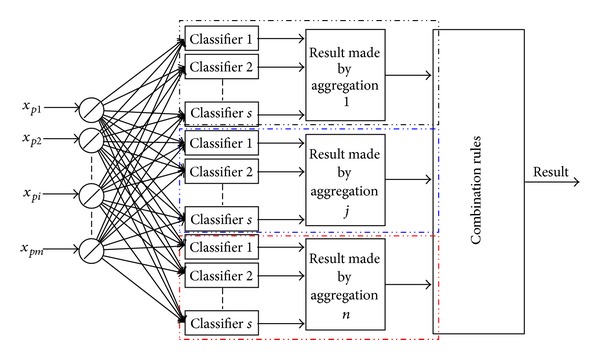
Multiple classifier aggregations to multiple faults classes.

Given *n* fault classes {*ω*_1_, *ω*_2_,…, *ω*_*j*_,…, *ω*_*n*_}, there will be *n* classifier aggregations in theory, and one aggregation refers to one classified result as shown in Figure 4.

Supposing an aggregation *j* is composed of *s* classifiers, to sample *x*_*p*_ ∈ *R* ^*m*^, the output of all classifiers in *j* is *y*_*p*_^(*j*)^ = (*y*_*p*1_^(*j*)^,…, *y*_*pk*_^(*j*)^,…, *y*_*ps*_^(*j*)^)^*T*^. For example, set a threshold *θ* as

(8) $$\begin{array}{c} \lambda_{jk}=\{\begin{array}{cc} 1 & \text{if}\;\;y_{p}^{\left(k\right)}\geq \theta \\ 0 & \text{otherwise} \end{array} \end{array}$$

and the majority vote rule can be described as

(9) $$\begin{array}{c} x_{\rho }\in \omega_{j}\;\text{if}\;\;\overset{s}{\underset{k=1}{\sum }}\lambda_{jk}=\underset{1\leq r\leq n}{\max}\overset{s}{\underset{k=1}{\sum }}\lambda_{rk}. \end{array}$$

It is said that for each class *ω*_*k*_ the sum on the right hand side of (9) simply counts the votes received for this hypothesis from the individual classifiers. The class which receives the largest number of votes is then selected as the consensus (majority) decision.

Take the XOR problem for example; the solution method using combining classifiers is as follows. The decision equations of three classifiers are

(10) $$\begin{array}{c} \pi_{1}:y=\left(-x_{1}+0.5\right)\cap \left(x_{2}+0.5\right),\\ \pi_{2}:y=\left(x_{1}-0.5\right)\cap \left(-x_{2}+0.5\right),\\ \pi_{3}:y=-\left(-x_{1}+0.5\right)\cap \left(-x_{2}+0.5\right). \end{array}$$

**Figure 5:**
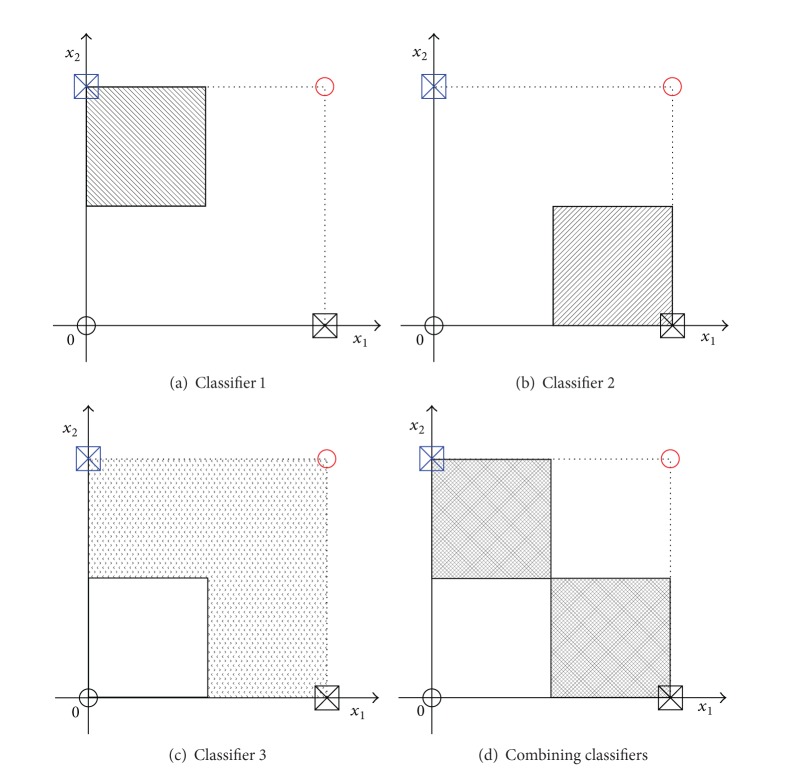
The XOR problem solved by combining classifiers.

The decision region is as shown in Figure 5. It shows that any classifier's accuracy is only 75% to this problem, but the accuracy will be 100% using combining classifiers with the majority vote rule. It is said that the classification accuracy of single classifier is low sometimes, but the accuracy will be improved greatly using combining classifiers.

Considering the fault associativity, there will be *m* fault models *M*_*i*_ (*i* = 1,2,…, *m*) (*m* is also the number of faults) trained by the data from related parameters set *P*_*i*_ for fault *f*_*i*_ using the second method mentioned in Section 3.2. Combining the *m* fault classifiers will not only improve the accuracy of model, but also cover all the fault classes.

## 4. Hybrid Voting Mechanism

Satellite fault diagnosis is a classical multiple faults problem. Its complexity depends on the fact that not only the types of faults are numerous, but also the number of parameters is large. Using SVM to diagnose satellite faults, there are two problems that need to be solved. The first problem is, to so many parameters, how to find the mapping relations between faults and parameters and reduce the interactions among them. Second, to multiple classifiers, the way of combining the results from them also needs to be considered. Considering the two problems, a multiple-model SVM based on a hybrid voting mechanism (HVM-SVM) is proposed, in which not only the combining classifiers are used to vote, but also the fault associativity is added to improve the voting. Obviously, in satellite fault diagnosis, when a fault occurs, only some of the parameters related to it are changed. That is to say, if some related parameters are abnormal, the fault may have happened.

Supposing a parameter set *p* = {*p*_*i*_,…, *p*_*j*_,…, *p*_*k*_} related to a fault *f*_*i*_, where *p* ∈ *R* ^*n*^, *R* includes all parameters, and *θ*_*i*_ is the threshold of *p*_*i*_. Defining a fault signature *δ*_*i*_ for *p*_*i*_ (the expected output and actual output of *p*_*i*_ are *d*_*i*_ and *y*_*i*_, resp.),

(11) $$\begin{array}{c} \delta_{i}=\{\begin{array}{cc} 1 & \text{if}\;\;\left|d_{i}-y_{i}\right|\geq \theta_{i}\\ 0 & \text{otherwise}. \end{array} \end{array}$$

Considering the interactions in the parameters, the essential condition of fault *f*_*i*_ which occurred is defined as

(12) $$\begin{array}{c} C=\frac{\delta_{i}+\delta_{j}+\cdots +\delta_{k}}{||p||}>0. \end{array}$$

HVM-SVM is a new combined strategy based on SVM. It generated multiple fault models using SVM to learn all fault data with the data of related parameters set. Through combining the results from these models and the essential condition of faults to the new data, HVM-SVM will obtain higher fault recognition performance. In the combination, due to different fault models with the different contribution to the fault diagnosis, how to set the weight of each single SVM classifiers is very important. A popular solution is based on the classification error rate to assign the weight of each classifier [15]. Assuming the classification error rate of the *M*_*i*_ model is *q*_*i*_, the weight *w*_*i*_ of the model can be defined as follows:

(13) $$\begin{array}{c} w_{i}=\log\frac{1-q_{i}}{q_{i}}. \end{array}$$

The HVM-SVM algorithm is divided into two stages:* fault feature extraction* and* model training*. The feature extraction is to establish the parameters set *p* related to the faults, and it can be obtained by the parameter reduction algorithm mentioned in Section 3.1. Considering the fault *f*_*i*_ and the red(*P*) = *P*_*i*_, model *M*_*i*_ is trained and only used the data of *P*_*i*_ so that it can reduce the bad influence from the unrelated parameters in the model training. The algorithms for model training are as in Algorithm 2.

For multiple faults, the multiple fault models *g*(*i*) will generate for each fault *f*_*i*_ using the relation data of red(*P*) = *P*_*i*_. Defining a decision function *D*(*i*, *j*) of the *j*th data record using *g*(*i*), in which it returns the classification result of the data record,

(14) $$\begin{array}{c} D\left(i,j\right)={\left(v_{1},v_{2},\ldots ,v_{m}\right)}^{T},\\ v_{i}=\{\begin{array}{cc} 1, & \text{if}\;\;\text{the}\;\;\text{result}\;\;\text{of}\;\;g\left(i\right)\;\text{is}\;\;f_{i}\\ 0, & \text{else}. \end{array} \end{array}$$

**Figure 6:**
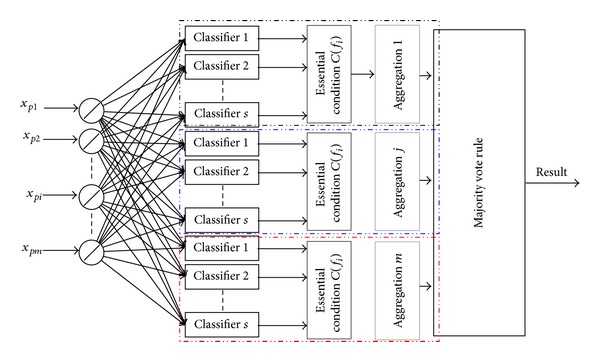
Combining SVM classifiers based on hybrid voting mechanism.

The function *D*(*i*, *j*) is only the decision from the model *g*(*i*), and the essential condition of fault *f*_*i*_ from the relation parameters set *p* is also considered in HVM-SVM, which is also shown in Figure 6.

So a vector to describe the essential condition of fault *f*_*i*_ is defined as follows:

(15) $$\begin{array}{c} C\left(f_{i}\right)={\left(x_{1},x_{2},\ldots ,x_{m}\right)}^{T},\\ x_{i}=\{\begin{array}{cc} \frac{\delta_{i}+\delta_{j}+\cdots +\delta_{k}}{||p||}, & \text{if}\;\;\text{the}\;\;\text{result}\;\;\text{of}\;\;g\left(i\right)\;\text{is}\;\;f_{i}\\ 0, & \text{else}. \end{array} \end{array}$$

And the hybrid decision function using the majority vote rule for multiple faults diagnosis is as follows:

(16) $$\begin{array}{c} V_{j}=\max\left(\overset{m}{\underset{i=1}{\sum }}\left(D\left(i,j\right)*w\left(i\right)+C\left(f_{i}\right)\right)\right). \end{array}$$

The algorithm for multiple faults model is described as in Algorithm 3.

## 5. Experimental Evaluation

Many experiments have been tested to evaluate the accuracy of HVM-SVM from single fault to multiple faults. For multiple faults diagnosis, the SVM, *k*-nearest neighbor, and neural network are selected to compare with HVM-SVM. The satellite remote sensing data is chosen as the test data, including the normal data and the fault data.

### 5.1. Single-Fault Model

In the first experiment, we assume that only one fault exists in the satellite system. The fault is defined as fault *A*, and the rest of the data is normal, defined as status *N*. The *p*_*A*_ = {*p*_*i*_,…, *p*_*j*_,…, *p*_*k*_} is a parameters set related to fault *A*. First, the relevant parameters are not considered for SVM to training diagnosis model. We used parts of the data as training samples including all the parameters and the rest of the data as testing samples. Two evaluation indexes are assigned to measure the accuracy of fault diagnosis: false alarm rate (FAR) and false negative rate (FNR):

(17) $$\begin{array}{c} \text{false}\;\;\text{alarm}\;\;\text{rate}=\frac{\text{FA}}{\text{FA}+\text{TN}},\\ \text{false}\;\;\text{negative}\;\;\text{rate}=\frac{\text{FN}}{\text{FN}+\text{TF}}. \end{array}$$

**Figure 7:**
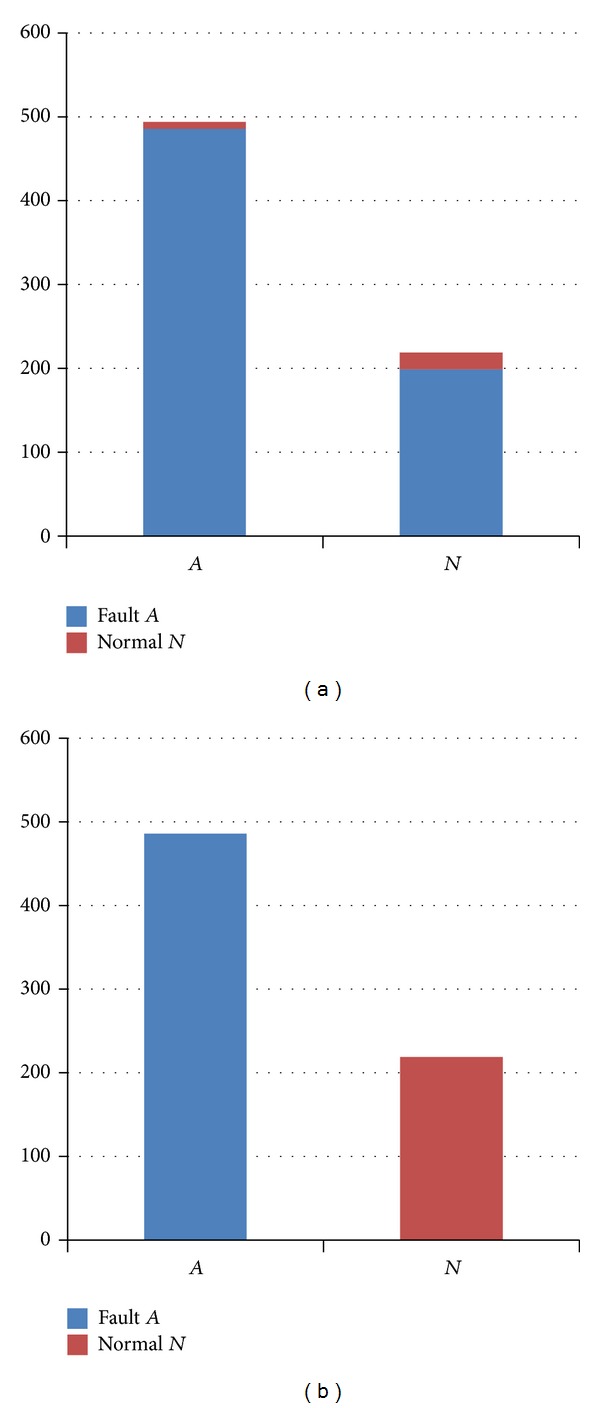
(a) The fault diagnosis results not considering the related parameters. (b) The ideal results with 100% accuracy.

FA is the number of false alarms, and TN is the number of true negatives. FN is the number of false negatives, and TF is the number of true faults. The experimental results are as shown in Figure 7.

**Figure 8:**
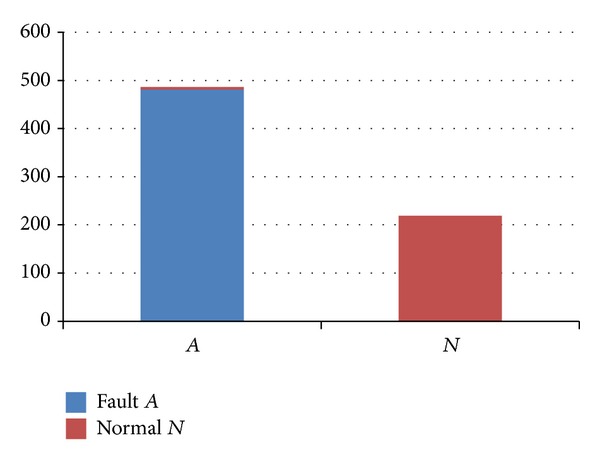
The diagnosis results considering the related parameters.

In Figure 7, it can be seen that the FAR = 8/(8 + 478) = 0.016, but the FNR is 199/(199 + 20) = 0.91. It is said that more than 90% of normal data are diagnosed as fault *A*. The reason is that the *A*'s diagnosis model is training using the whole parameters, not considering the parameter reduction, which caused the inaccuracy and instability of fault model. In order to reduce the interactions from other irrelevant parameters, we only used the parameters in *p*_*A*_ to train the diagnosis model of fault *A*. The results of this model are shown in Figure 8.

### 5.2. Multiple Faults Model

**Figure 9:**
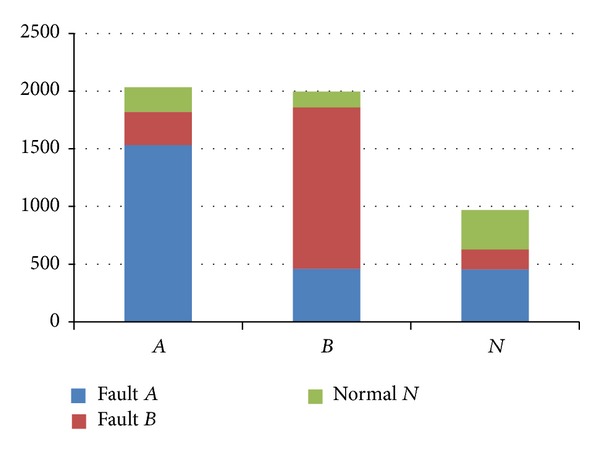
The results using single model to classify multiple faults.

It is obvious that the satellite fault diagnosis is not a single-fault problem. Suppose there are three modes in satellite system: fault *A*, fault *B*, and normal *N*. The related parameters are also considered, and the training samples are composed of *p*_*A*_ and *p*_*B*_, respectively. Using the second method of SVM introduced in Section 3.2, a simple multiple faults model can be obtained. Using the model to diagnose the fault, the results are as shown in Figure 9.

It is can be seen from Figure 9 that using single model to classify faults is not a good way, and the FAR of each class will be high. In the above example, the FARs are 24.6%, 30%, and 64.6%, respectively.

**Figure 10:**
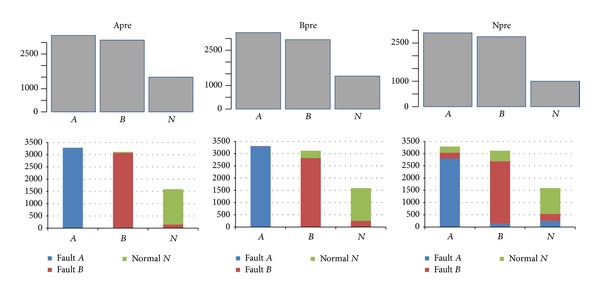
Using three models to diagnose the satellite remote sensing data, respectively.

For multiple faults, the single model will get the bad performance. Next, the HVM-SVM is used to diagnose the multiple faults, and the same data is used but it added more records. There are three models *M*_*A*_, *M*_*B*_, and *M*_*N*_ trained from related data, that is, *M*_*A*_ obtained from *p*_*A*_, *M*_*B*_ obtained from *p*_*B*_, and *M*_*N*_ obtained from the normal data. Using the three models to diagnose the satellite data, respectively, the results are shown in Figure 10.

It can be seen from Figure 10 that each model has its own classification results for three modes, which is the best. We used the hybrid voting mechanism of HVM-SVM to integrate the results. The results can be seen in Table 2.

It is said that, after four votes (three by classifiers and one by essential condition of faults), there are three records misclassified as fault *B* in fault *A*, 11 misclassification records in fault *B*, and 33 error records in normal *N*. The fault recognition rate of *A*, *B*, and *N* is 99.9%, 99.6%, and 97.9%, respectively.

### 5.3. The Accuracy of HVM-SVM

**Figure 11:**
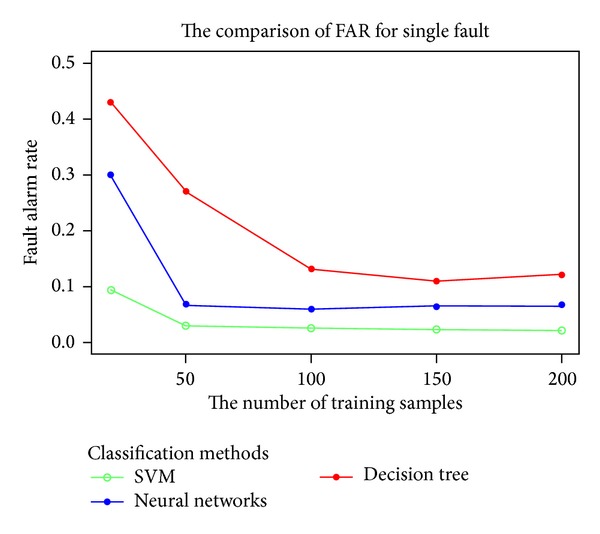
The FAR of three methods with small fault samples.

HVM-SVM used combining classifiers strategy to improve the accuracy of fault diagnosis. In fact, there are many classification methods which can be used as the classifiers, such as neural networks, decision trees, *k*-nearest neighbor, and naive Bayes. Why is only SVM used for HVM-SVM? As mentioned in Section 1, SVM is very suitable for satellite faults diagnosis duo to its small fault samples. The following experiment can demonstrate the fact. The SVM, neural networks, and decision tree are selected to test the FAR on small fault samples. It can be seen from Figure 11 that when the number of training samples becomes smaller, the FAR of SVM is significantly lower than the other two methods.

The performance of HVM-SVM is measured by the accuracy rate of multiple faults diagnosis compared with SVM, neural networks, and *k*-nearest neighbor. The experimental data and fault types are the same with Section 5.2. Two evaluation indexes are assigned to measure the accuracy of multiple faults diagnosis: fault recall rate (FRR) and fault *x* misclassified as fault *y* (FAR(*x* → *y*)):

(18) $$\begin{array}{c} \text{fault}\;\;\text{recall}\;\;\text{rate}=\frac{\text{FA}}{\text{TF}},\\ \text{FAR}\left(x⟶y\right)=\frac{\text{the}\;\;\text{number}\;\;\text{of}\;\;x\;\;\text{misclassified}\;\;\text{as}\;\;y}{\text{TF}\;\;\text{of}\;\;x}. \end{array}$$

The experimental results are shown in Table 3.

**Figure 12:**
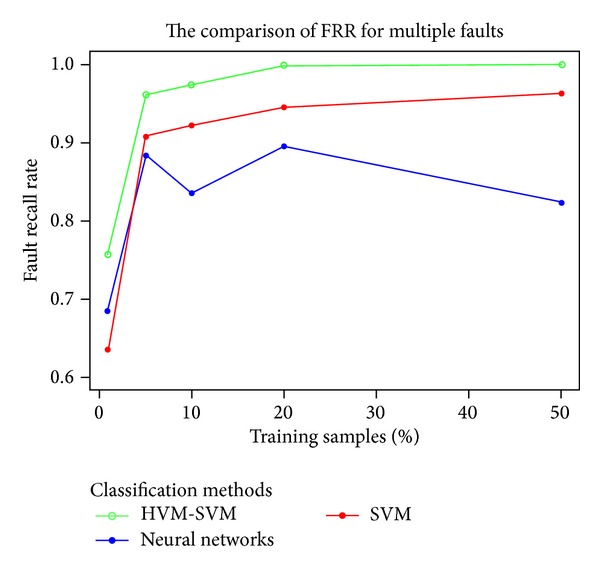
The FRR of three methods to multiple faults diagnosis.

From Table 3, HVM-SVM gets the best performance in the multiple faults diagnosis, especially when the percentage of training samples plunged to 1%; it still has almost 70% accuracy rate for separating fault *A*. Most methods will be more precise with the training samples increased, but the neural networks have a little instability in accuracy. Although SVM will get higher accuracy for small samples, to multiple faults diagnosis, it may misclassify faults with high probability. The comparison of the three methods in FRR is also illustrated in Figure 12.

## 6. Conclusion

The satellite fault diagnosis is different from general fault diagnosis for its special features, such as enormous parameters, multiple faults, and small samples. The limitations and complexity of space environment make the problem more serious. In this paper, we introduced an improving SVM algorithm based on a hybrid voting mechanism to enhance the accuracy of satellite fault diagnosis. To reduce the interactions of multiple parameters, we proposed a parameter reduction algorithm to find the mapping relation between faults and the parameters. SVM is suitable for classifying small samples, but not multiple faults. We combine multiple SVM classifiers and use the majority vote rule to deal with multiple faults. The contribution of our method is that not only the combining classifiers are used to vote, but also the fault associativity (fault essential condition) is added to improve the voting. Many experimental results illustrated that the HVM-SVM method is very suitable for satellite fault diagnosis and, compared to some classification methods, it has the best performance.

## Figures and Tables

**Algorithm 1:**
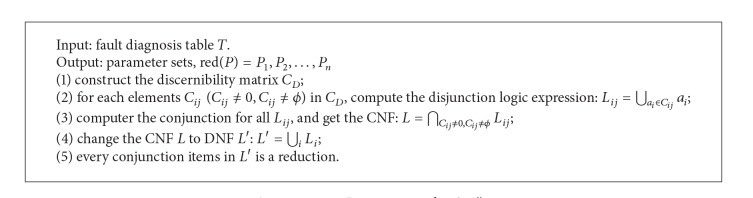
*Parameter*_*reduction*().

**Algorithm 2:**
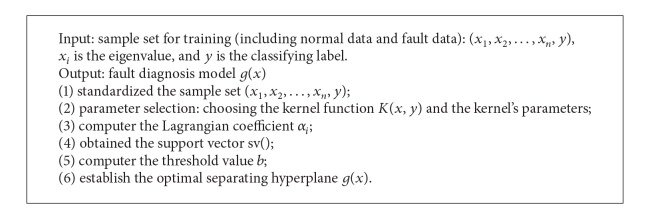
*Single*_*Fault*().

**Algorithm 3:**
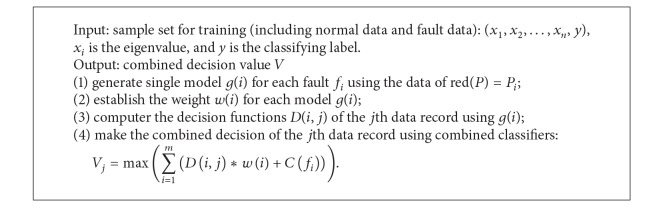
*Multiple*_*Fault*().

**Table 1:** A fault diagnosis table.

*U* ^Φ^	*f*(*a*)	*f*(*b*)	*f*(*c*)	*f*(*d*)	*f*(*e*)	Faults
1	0	1	0	1	1	*A*
2	1	0	1	0	1	*B*
3	0	1	0	0	1	*C*
4	0	0	0	0	1	*D*
5	0	1	1	0	1	*E*
6	0	1	1	1	1	*F*

**Table 2:** Integrate the results using hybrid voting mechanism.

	Fault *A*	Fault *B*	Normal *N*
*A*	3288	8	3
*B*	3	3106	30
*N*	0	3	1559

**Table 3:** The FRR and FAR of HVM-SVM compared with SVM, NN, and KNN (only list parts of the results).

A total of 10000 records		5000 for training 5000 for testing	2000 for training 8000 for testing	1000 for training 9000 for testing	500 for training 9500 for testing	100 for training 9900 for testing
SVM	FRR (fault *A*)	0.9990301	0.9981768	0.9902465	0.9897119	0.006655164
	FAR (*A* → *B*)	0.00048497	0.00060772	0.005689515	0.00617284	0.9933448
	FAR (*A* → *N*)	0.0004849	0.00121544	0.004063939	0.004115226	0

HVM-SVM	FRR (fault *A*)	1	0.9990884	0.9929558	0.9927984	0.6958344
	FAR (*A* → *B*)	0	0.00091158	0.00270929	0.00308642	0.3041653
	FAR (*A* → *N*)	0	0	0.00379301	0.00360082	0

NN	FRR (fault *A*)	0.8962306	0.9541473	0.9788487	0.9405237	0.6340591
	FAR (*A* → *B*)	0.1037694	0.04585265	0.0111413	0.01945227	0.1875932
	FAR (*A* → *N*)	0	0	0.01001	0.040024	0.1783477

KNN	FRR (fault *A*)	0.9960396	0.9792714	0.9149528	0.8333333	0.4125697
	FAR (*A* → *B*)	0.0039603	0.00188442	0.00166759	0.00157729	0.204764
	FAR (*A* → *N*)	0	0.01884422	0.0794886	0.139327	0.382666
